# Calreticulin expression and localization in relation to exchangeable Ca^2+^ during pollen development in *Petunia*

**DOI:** 10.1186/s12870-021-03409-4

**Published:** 2022-01-08

**Authors:** Anna Suwińska, Piotr Wasąg, Elżbieta Bednarska-Kozakiewicz, Marta Lenartowska, Robert Lenartowski

**Affiliations:** 1 Department of Cellular and Molecular Biology, Faculty of Biological and Veterinary Sciences, Nicolaus Copernicus University in Toruń, Toruń, Poland; 2 Centre for Modern Interdisciplinary Technologies, Nicolaus Copernicus University in Toruń, Toruń, Poland; 3 Department of Biochemistry and Cell Biology, Faculty of Biological Sciences, Kazimierz Wielki University, Bydgoszcz, Poland

**Keywords:** Anther, Calcium homeostasis, Calreticulin, Gene expression, Immunocytochemistry, Microsporo/gametogenesis, Molecular chaperoning, *Petunia hybrida*, Pollen development

## Abstract

**Background:**

Pollen development in the anther in angiosperms depends on complicated cellular interactions associated with the expression of gametophytic and sporophytic genes which control fundamental processes during microsporo/gametogenesis, such as exo/endocytosis, intracellular transport, cell signaling, chromatin remodeling, and cell division. Most if not all of these cellular processes depend of local concentration of calcium ions (Ca^2+^). Work from our laboratory and others provide evidence that calreticulin (CRT), a prominent Ca^2+^-binding/buffering protein in the endoplasmic reticulum (ER) of eukaryotic cells, may be involved in pollen formation and function. Here, we show for the first time the expression pattern of the *PhCRT1* gene and CRT accumulation in relation to exchangeable Ca^2+^ in *Petunia hybrida* developing anther, and discuss probable roles for this protein in the male gametophyte development.

**Results:**

Using northern hybridization, western blot analysis, fluorescent in situ hybridization (FISH), immunocytochemistry, and potassium antimonate precipitation, we report that *PhCRT1* is highly expressed in the anther and localization pattern of the CRT protein correlates with loosely bound (exchangeable) Ca^2+^ during the successive stages of microsporo/gametogenesis. We confirmed a permanent presence of both CRT and exchangeable Ca^2+^ in the germ line and tapetal cells, where these factors preferentially localized to the ER which is known to be the most effective intracellular Ca^2+^ store in eukaryotic cells. In addition, our immunoblots revealed a gradual increase in CRT level from the microsporocyte stage through the meiosis and the highest CRT level at the microspore stage, when both microspores and tapetal cells show extremely high secretory activity correlated with the biogenesis of the sporoderm.

**Conclusion:**

Our present data provide support for a key role of CRT in developing anther of angiosperms – regulation of Ca^2+^ homeostasis during pollen grains formation. This Ca^2+^-buffering chaperone seems to be essential for pollen development and maturation since a high rate of protein synthesis and protein folding within the ER as well as intracellular Ca^2+^ homeostasis are strictly required during the multi-step process of pollen development.

**Supplementary Information:**

The online version contains supplementary material available at 10.1186/s12870-021-03409-4.

## Background

**Fig. 1:**
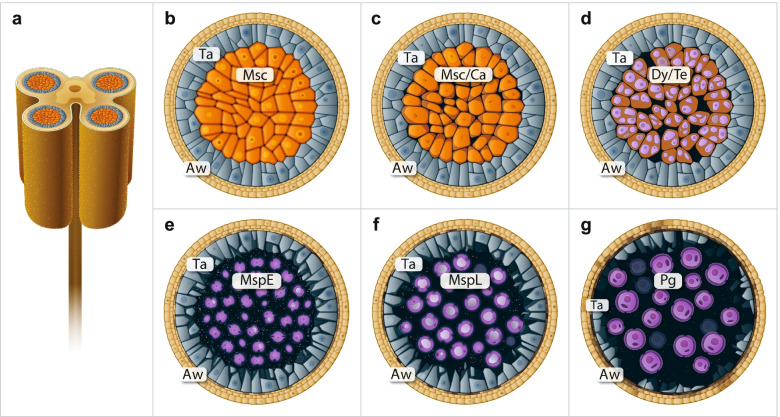
Scheme of microsporo/gametogenesis in angiosperms. *Cartoons* represent cross sections of the tetrasporangiate *Petunia* anther (**a**), microsporocyte (*Msc*) stage (**b**), callose (*Msc/Ca*) stage (**c**), dyad/tetrad (*Dy/Te*) stage (**d**), early microspore (*MspE*) stage (**e**), late microspore (*MspL*) stage (**f**), and pollen grain (*Pg*) stage (**g**). *Aw* anther wall, *Ta* tapetum

In angiosperms, pollen grain represents a highly reduced male gametophyte consisting of just two or three cells, when mature pollen is released from the anther. Pollen development is a multi-stage process, usually divided into two main phases: microsporogenesis that comprises formation of microspores during meiosis, and microgametogenesis that involves progressive transformation of microspores into mature pollen grains, followed by the growth of a pollen tube containing two sperm cells (male gametes). During microsporogenesis (Fig. 1a-f), diploid microsporocytes undergo meiotic division (Fig. 1b-c) to produce dyads, and then tetrads of haploid microspores (Fig. 1d) which are surrounded by a specific callosic cell wall. This stage is completed when unicellular microspores are released from the tetrads (Fig. 1e-f) by activity of callase enzyme secreted by tapetum, the nutritive tissue in the anther. Then, microgametogenesis begins; each microspore undergoes an asymmetric cell division giving two differential cells of the pollen grain (Fig. 1g). Much bigger and metabolically active vegetative cell accumulates carbohydrates, lipids, numerous transcripts and proteins that are required for rapid pollen tube growth during compatible pollination [1]. By contrast, definitely smaller generative cell has highly condensed nuclear chromatin and continues through a further round of mitosis to produce two sperm cells. In species that produce tricellular pollen grains, the second pollen mitosis takes place in the maturing anther. The majority of dicotyledonous plants (such as *Petunia*) shed their pollen in a bicellular state (Fig. 1g) and the generative cell divides into two sperm cells in growing pollen tube.

Differences between the successive stages of pollen development are reflected in their transcriptomic profiles. For example, in different plant species more genes are expressed in microspores and bicellular pollen but their number progressively declines in mature pollen [2]. Although there is significant overlap with the majority of genes expressed in these phases of microsporo/gametogenesis, the percentage of specific genes increases from unicellular microspore stage to mature pollen. The reduction in complexity and switch to a late program – pollen maturation – is also accompanied by an increase in expression of genes involved in cell wall metabolism, cytoskeleton functions, and cell signaling [2–4]. Biogenesis of the multilayer pollen cell wall (sporoderm) starts from the tetrad stage and requires intense protein synthesis correlated with protein folding and extremely high secretory activity of the microspores and maturing pollen grains, and the tapetal cells. Progress of the microsporo/gametogenesis depends also on complicated cellular interactions associated with tissue- and cell-specific expression of gametophytic and sporophytic genes which control fundamental cellular processes during pollen formation, such as exo/endocytosis, intracellular transport, cell signaling, chromatin remodeling, and cell division. Most if not all of these processes depend of local concentration of Ca^2+^ [4–6].

Work from our laboratory and others provide evidence that CRT, a prominent Ca^2+^-binding/buffering protein in the ER of eukaryotic cells [7] may be involved in pollen formation and function. The plant CRT family consists of three members: CRT1, CRT2 and CRT3; while CRT1/2 isoforms represent one subgroup and appear to work as primary proteins within a general ER chaperone framework, plant-specific CRT3 is a divergent member that seems to be co-expressed with pathogen/signal transduction-related genes [8–10]. CRT and/or its mRNAs were found in dry and germinating pollen/tubes of *Ginkgo* [11], *Liriodendron* [12], *Nicotiana* [13], *Haemanthus* [14], *Arabidopsis* [15] and *Petunia* [16–20]. But to date, only three reports proved CRT expression or localization in developing anther of angiosperms. First, CRT and CRT mRNAs were detected in both the tapetal and germline cells in *Nicotiana* anther [13]. Second, high expression of specific *CRT* genes (*CRT1/2* but not *CRT3*) was confirmed in *Arabidopsis* developing and mature anthers [15]. Third, involvement of CRT was suggested in formation of tapetosomes during pollen development in *Brassica* [21]. In plants (as in animals) this multifunctional chaperone participates in numerous cellular processes, such as gene expression, cell signaling, apoptosis, and cell-to-cell communication by plasmodesmata through its two main functions: Ca^2+^ storage/mobilization and protein folding [7, 9]. Both these functions seem to be essential for pollen development and maturation since a high rate of RNA/protein synthesis and intracellular Ca^2+^ homeostasis are strictly required during the multi-step processes of microsporo/gametogenesis.

Recently, we have cloned and characterized the full-length cDNA of the *PhCRT* gene (belonging to the subgroup *CRT1/2*) and showed its elevated expression during the key reproductive events in the pistil, when the level of exchangeable Ca^2+^ changes dynamically [17, 18]*.* We have also demonstrated that *Petunia* pollen tubes accumulate of both *PhCRT1/2* mRNA and CRT protein in the ER-rich cytoplasmic regions [19]. Moreover, we found that post-transcriptional silencing of *PhCRT1/2* expression impairs pollen tube elongation [20]. Thus, we proposed an important role for CRT belonging to the subgroup CRT1/2 during the key reproductive events in angiosperms. Here, we show for the first time the expression of *PhCRT1* gene and CRT accumulation in relation to exchangeable Ca^2+^ in *Petunia* developing anther, and discuss probable roles for this protein in the male gametophyte development.

## Results

### Expression pattern of the *PhCRT1* gene in developing anther

**Fig. 2:**
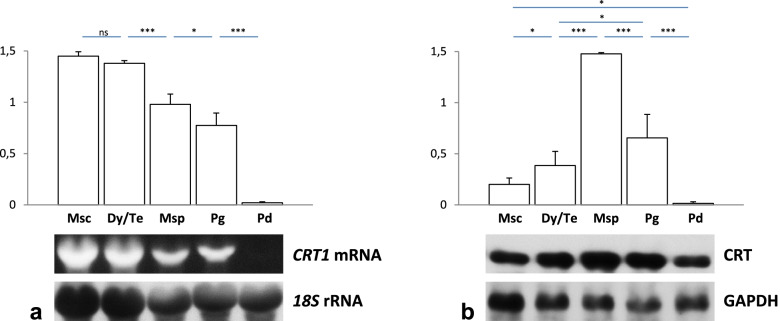
Northern blot analysis of *PhCRT1* mRNA transcript levels (**a**) and western blot analysis of CRT levels (**b**) in whole *Petunia* anthers during subsequent stages of pollen development (*Msc* microsporocyte, *Dy/Te* dyad/tetrad, *Msp* microspore, *Pg* pollen grain stages) and in dry pollen (*Pd*). **a** *Graphs* show the relative *PhCRT* mRNA levels (mean of three replicates and standard deviation) normalized to the *Ph18S* rRNA levels. Statistical analysis was carried out by one-way ANOVA (*ns* not significant, *P ≤ 0.05; **P ≤ 0.01; ***P ≤ 0.001). Cropped representative northern blots are shown under the *graphs*. Blots were hybridized with *PhCRT1* antisense probe, and then re-hybridized with *Ph18S* rRNA probe. **b** *Graphs* show the relative CRT levels (mean of three replicates and standard deviation) normalized to the *Petunia* glyceraldehyde-3-phosphate dehydrogenase (GAPDH) levels. Statistical analysis was carried out by one-way ANOVA ANOVA (*ns* not significant, *P ≤ 0.05; **P ≤ 0.01; ***P ≤ 0.001). Cropped representative western blots are shown under the *graphs*. Original northern and western blots are shown in the Fig. S1 (Supplementary information)

To analyze the expression pattern of *PhCRT1* in relation to subsequent stages of pollen development, we first investigated *PhCRT1* mRNA transcript levels at particular phases of microsporo/gametogenesis (microsporocytes, dyads/tetrads, microspores, pollen grains) and in dry pollen collected from mature anther at the anther dehiscence. Northern blot analysis of total *Petunia* anther RNA revealed that the highest level of *PhCRT1* expression was at the microsporocyte stage before meiosis (Fig. 2a). Expression then decreased gradually through the dyad/tetrad, microspore, and pollen grain stages, and dropped significantly in dry pollen. This result is consistent with the previous studies by [13] who reported that the high levels of *CRT* mRNA transcripts (undefined *CRT* gene) were detected throughout anther development in *Nicotiana*, while *CRT* expression was much lower in dry or hydrated pollen. This result indicates that the transcriptional activity of *PhCRT1* or the stabilization of *PhCRT1* mRNA decreases with progressive anther development and pollen maturation.

Next, we performed western blot analysis of total proteins isolated from whole *Petunia* anthers and dry pollen. Using the polyclonal antibody against CRT (CRT PAb) that we have previously demonstrated to be specific for CRT in *Petunia* [16], we detected significantly low level of CRT at the microsporocyte stage (Fig. 2b). However, we observed almost twofold increase in the protein level during meiosis and peak at the microspore stage, more than threefold higher level than in the dyad/tetrad stage. The highest level of CRT could be indicative of a response to biogenesis of the sporoderm when both microspores and tapetal cells show extremely high secretory activity correlated with protein synthesis and protein folding in the ER. Next, the CRT level decreased at the pollen grain stage, reaching its lowest value in dry pollen at the anther dehiscence. Again, this result is consistent with the previous studies in *Nicotiana* anther which showed a particularly dense CRT immunolabeling in developing pollen and in the active tapetum through tetrads to pollen grains, and much lower signal in dry and hydrated pollen [13]. Moreover, our in vitro hybridization and immunoblot results show that transcriptional activation of the *PhCRT1* gene occurs at the beginning of microsporogenesis, however, the whole pool of transcripts do not undergo translation at once, and do not contribute to increase in CRT level at the microsporocyte stage. Activation of translation (and, as a result significant CRT increase) starts with a slight delay reaching its highest level at the microspore stage what is correlated with biogenesis of the sporoderm.

### Spatiotemporal localization of the *PhCRT1* mRNA transcripts in developing anther

**Fig. 3:**
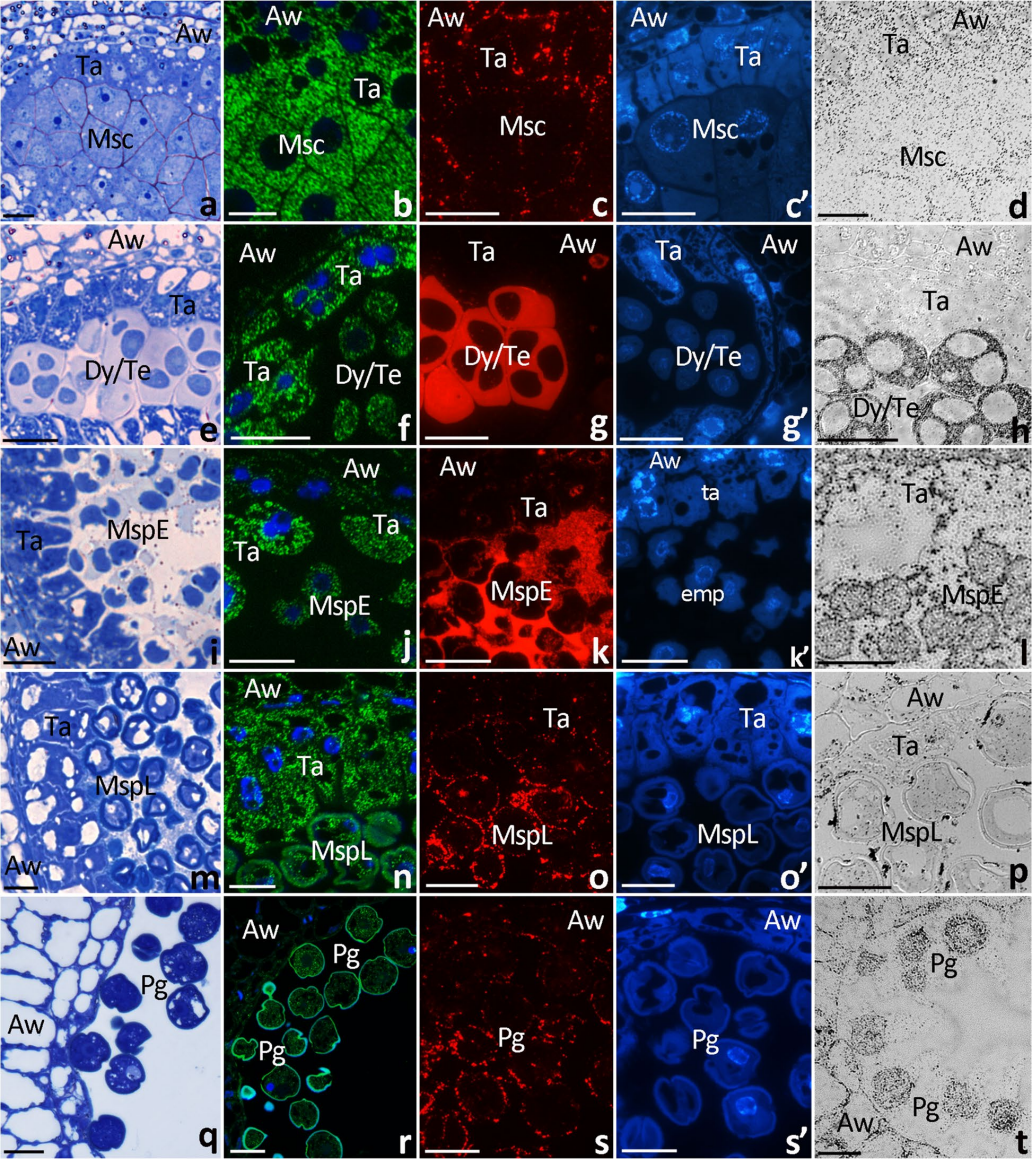
Cross sections via the single locule of *Petunia* anther show: methylene blue staining (**a**, **e**, **i**, **m**, **q**), localization of *PhCRT1* mRNA transcripts using FISH (*green*, **b**, **f**, **j**, **n**, **r**) and localization of CRT protein using immunofluorescence method (*red*, **c**, **g**, **k**, **o**, **s**) and immunogold-silver method (*black traces*, **d**, **h**, **l**, **p**, **t**) during subsequent stages of pollen development (*Msc* microsporocyte, *Dy/Te* dyad/tetrad, *MspE* early microspore, *MspL* late microspore, *Pg* pollen grain stages). Nuclei are stained by Hoechst 33342 (*blue*, **c’**, **g’**, **k’**, **o’**, **s’**). *Aw* anther wall, *Ta* tapetum. *Scale bars* correspond to 15 μm (**a**-**d**), 20 μm (**e**-**p**), and 25 μm (**q**-**t**)

To determine distribution of *PhCRT1* mRNAs in developing pollen and the tapetal cells, samples of anther dissected from flowers buds or open flowers were fixed, embedded, sectioned, processed for FISH and visualized by fluorescence microscopy. Several anther developmental stages were selected for FISH using standard methylene blue staining of semi-thin sections: diploid microsporocytes (Fig. 3a), dyads/tetrads (meiotic division, Fig. 3e), early microspores (haploid cells released from tetrads, Fig. 3i), late microspores (highly vacuolated cells, Fig. 3m), and maturing pollen grains (Fig. 3q). Cross sections of the anther before meiosis showed accumulation of *PhCRT1* mRNA transcripts in the cytoplasm of both microsporocytes and somatic tapetal cells (Fig. 3b). A comparable green fluorescence signal was also found in the cell cytoplasm of the anther wall. During meiotic division, when meiocytes form dyads and then tetrads, *PhCRT1* mRNA transcripts were still accumulated in the cytoplasm of the germ line and tapetal cells (Fig. 3f). However, at this stage the FISH signal was weaker in meiocytes than in tapetum. Similar localization patterns were observed after meiosis, when hybridization signals were detected predominantly in the cytoplasm of the tapetal cells, whereas both early and late microspores showed weaker labeling (Fig. 3j, n). Somatic cells of the anther wall were positive for *PhCRT1* mRNA through the dyad/tetrad stage to the late microspore stage, however, hybridization signals were only detected in the cytoplasm along the edge of these highly vacuolated cells (Fig. 3f, j, n). By contrast, maturing pollen grains showed uniform fluorescence throughout the cytoplasm except vacuoles (Fig. 3r). At this stage, very little FISH signals were detected in the anther wall cells, and no labeling was observed in degenerating tapetum. To gain insight into possible differential transcriptional activity of *PhCRT1,* we paid attention to FISH signals in the nuclei, as these may indicate nascent transcription. The nuclei of the germ line and somatic cells were all positive for *PhCRT1* mRNA, however, the transcripts were associated mainly with the nuclei of the tapetal cells at the early and late microspore stages (Fig. 3n). A very weak fluorescence was associated with the vegetative nuclei and no hybridization signals were detected in the generative nuclei of maturing pollen (Fig. 3r). Also vacuoles (Fig. 3f, j, n, r) and the callosic cell wall surrounding dyads/tetrads (Fig. 3f) were negative for *PhCRT1* mRNA. No labeling was observed when the probe was omitted (data not shown).

These results are consistent with our northern blot hybridization and confirm that the highest accumulation of *PhCRT1* mRNA transcripts in developing anther correlates with the microsporocyte stage (Fig. 2a), when both the germ line and somatic cells are highly active. As the microsporo/gametogenesis progress, the transcriptional activity of various anther cells changes. For example, meiotic division silences temporary this activity in germ line cells, while tapetum seems to be transcriptionally active during microsporogenesis up to the late microspore stage, and then degenerates. In addition, the FISH signal was significantly lower in pollen grains than in microsporocytes, dyads, tetrads or microspores, and a gradual disappearance of the fluorescence was observed also in the cells of the anther wall throughout pollen development.

### Spatiotemporal distribution of CRT during microsporo/gametogenesis

We next wished to compare localization of CRT in pollen chambers at selected stages of pollen development. To do this, we investigated *Petunia* anthers processed for immunofluorescence and immunogold-silver labeling. As shown in semi-thin sections stained with CRT PAb and the secondary antibodies, we confirmed presence of CRT in both the germ line and somatic cells of the anther, however the level of signal varied at different developmental stages and in different cells (Fig. 3c-d, g-h, k-l, o-p, s-t). Before meiosis, the labeling was evident in the cytoplasm of the microsporocytes, tapetum, and the anther wall cells, wherein in microsporocytes CRT was preferentially localized to the cortical cytoplasm (Fig. 3c-d). At the dyad/tetrad stage, enrichment of CRT was mainly observed extracellularly, because the red fluorescence (Fig. 3g) or silver traces (Fig. 3h) corresponded mainly with the thick cell wall that surrounded meiocytes. By contrast, the somatic cells – tapetal and the anther wall cells – showed much weaker immunolabeling than dyads and tetrads (Fig. 3g-h). In germ line cells, the strongest signals detected were associated with the early microspores (Fig. 3k-l). At this stage, enrichment of CRT was also observed in tapetal cells as well as extracellularly, between the early microspores in the anther locule. This result is consistent with our western blot analysis and confirm that the highest accumulation of CRT in developing anther correlates with the microspore stage (Fig. 2b), when both germ line and tapetal cells exhibit an extremely high metabolic activity correlated with the sporoderm biosynthesis. Next, we observed definitely weaker immunolabeling signal at the late microspore and the pollen grain stages (Fig. 3o-p and s-t, respectively). We confirmed presence of CRT in the cytoplasm of highly vacuolated late microspores and between these cells (Fig. 3o). Moreover, using the immunogold-silver technique we was able to distinguish CRT localization in apertures of the late microspores (Fig. 3p). Finally, CRT was detected in the cytoplasm of maturing pollen (Fig. 3s-t). At these two last stages of pollen development, very little immunocytochemical signals were detected in the somatic cells of the anther (Fig. 3o-p and s-t). Again, these results are consistent with our immunoblot analysis that showed a gradual decrease of the CRT level from the microspore stage to dry pollen (Fig. 2b).

### Subcellular localization of CRT with respect to exchangeable Ca^2+^

Given that CRT is able to bind Ca^2+^ reversibly, we next sought to determine the subcellular localization of the protein in relation to exchangeable Ca^2+^ at the selected stages of pollen development. To do so, we performed immuno-electron microscopy of CRT correlated with localization of loosely bound Ca^2+^ by potassium antimonate precipitation.

**Fig. 4:**
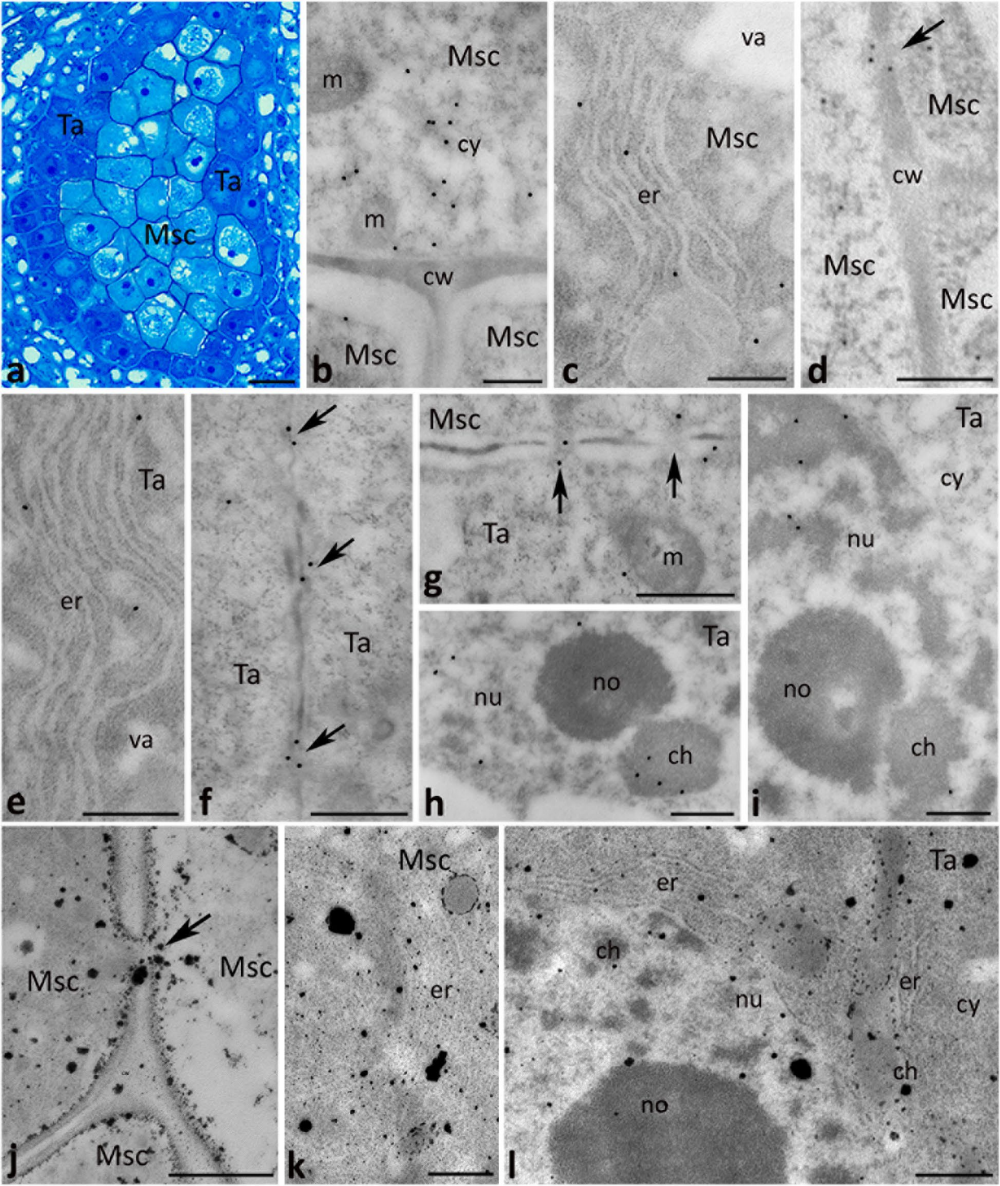
Cross sections via the single locule of *Petunia* anther at the microsporocyte stage (*Msc*) show: methylene blue staining (**a**), immunogold localization of CRT protein (**b**-**i**) and localization of exchangeable Ca^2+^ by potassium antimonite precipitation (**j**-**l**). *Arrows* in **d**, **f**, and **g** show immunolabeling associated with plasmodesmata, and *arrows* in **j** shows Ca^2+^ ppts associated with plasmodesmata. *ch* chromatin, *cw* cell wall, *cy* cytoplasm, *er* endoplasmic reticulum, *m* mitochondrium, *no* nucleolus, *nu* nucleus, *Ta* tapetum cell, *va* vacuole. *Scale bars* correspond to 15 μm (**a**) and 500 nm (**b**-**l**)

At the microsporocyte stage (Fig. 4a), CRT was detected in the cytoplasm of diploid microsporocytes (Fig. 4b) where the protein was typically localized to the ER (Fig. 4c). Epitopes binding CRT PAb were also found at plasmodesmata connecting adjacent microsporocytes (Fig. 4d, arrow). A similar CRT labeling pattern was revealed in the cytoplasm of the tapetal cells; gold traces were localized in the ER (Fig. 4e) and plasmodesmata connecting adjacent tapetal cells (Fig. 4f, arrows), and microsporocytes with the tapetal cells (Fig. 4g, arrows). Moreover, CRT was detected in the tapetal cell nuclei (Fig. 4h, i). With the immunogold technique, we were able to distinguish the protein localization in defined nucleus sub-domains, such as perichromatin areas (Fig. 4i) and the nucleous-associated chromatin (Fig. 4h). Consistent with CRT being a Ca^2+^-binding/buffering protein, Ca^2+^ precipitates (Ca^2+^ ppts) corresponding to exchangeable Ca^2+^ were observed in the same localizations where CRT was found; there were plasmodesmata (Fig. 4j, arrow) and the ER in both microsporocytes (Fig. 4k) and the tapetal cells (Fig. 4l). Several Ca^2+^ ppts were also detected in the chromatin of the tapetal cell nuclei (Fig. 4l).

**Fig. 5:**
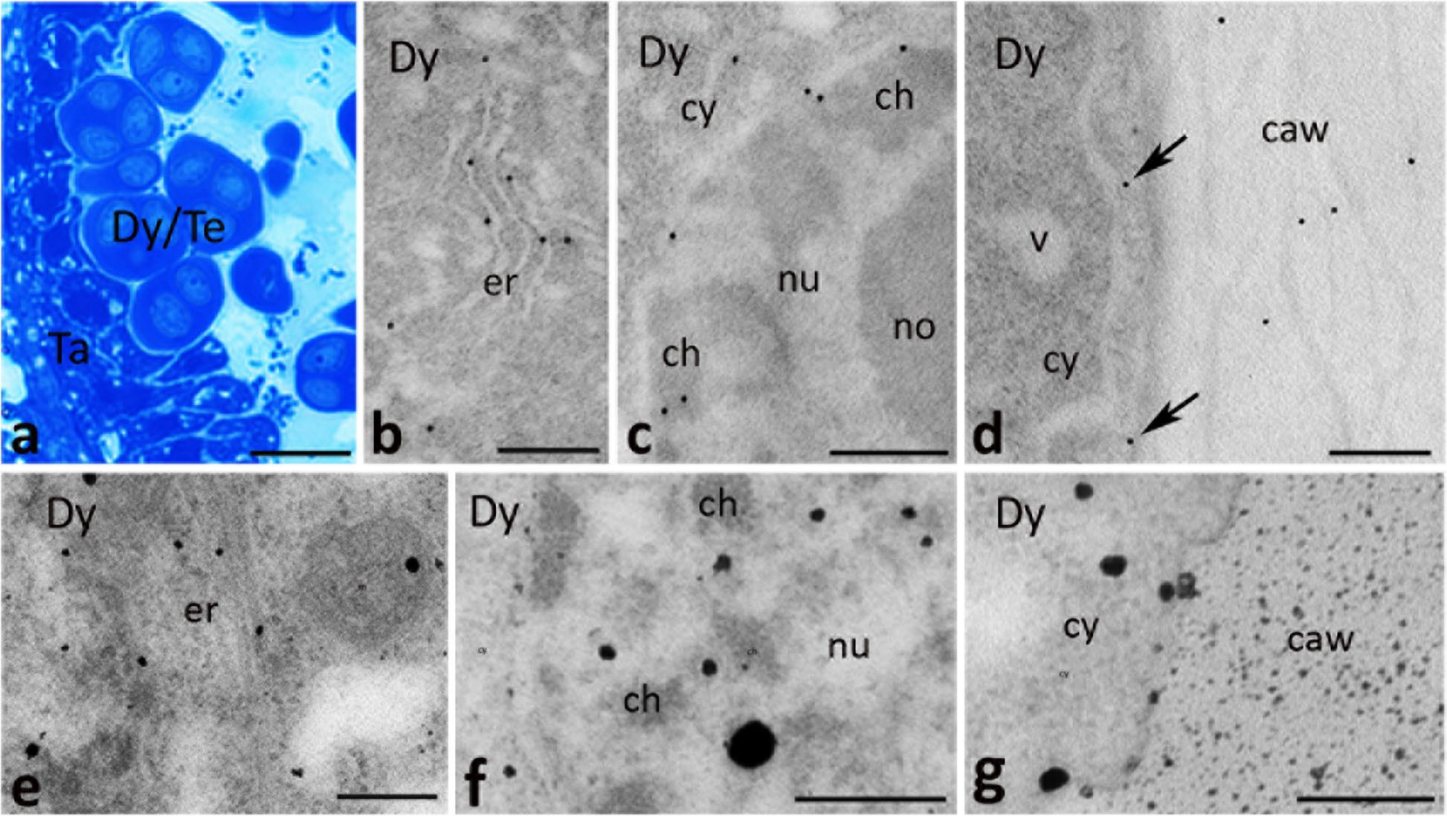
Cross sections via the single locule of *Petunia* anther at the dyad/tetrad stage (*Dy/Te*) show: methylene blue staining (**a**), immunogold localization of CRT protein (**b**-**d**) and localization of exchangeable Ca^2+^ by potassium antimonite precipitation (**e**-**g**). *Arrows* in **d** show immunolabeling at cell peripheries of meiocyte close to callosic cell wall (*caw*). *ch* chromatin, *cy* cytoplasm, *er* endoplasmic reticulum, *no* nucleolus, *nu* nucleus, *Ta* tapetum cell, *v* vesicle. *Scale bars* correspond to 20 μm (**a**) and 500 nm (**b**-**g**)

**Fig. 6:**
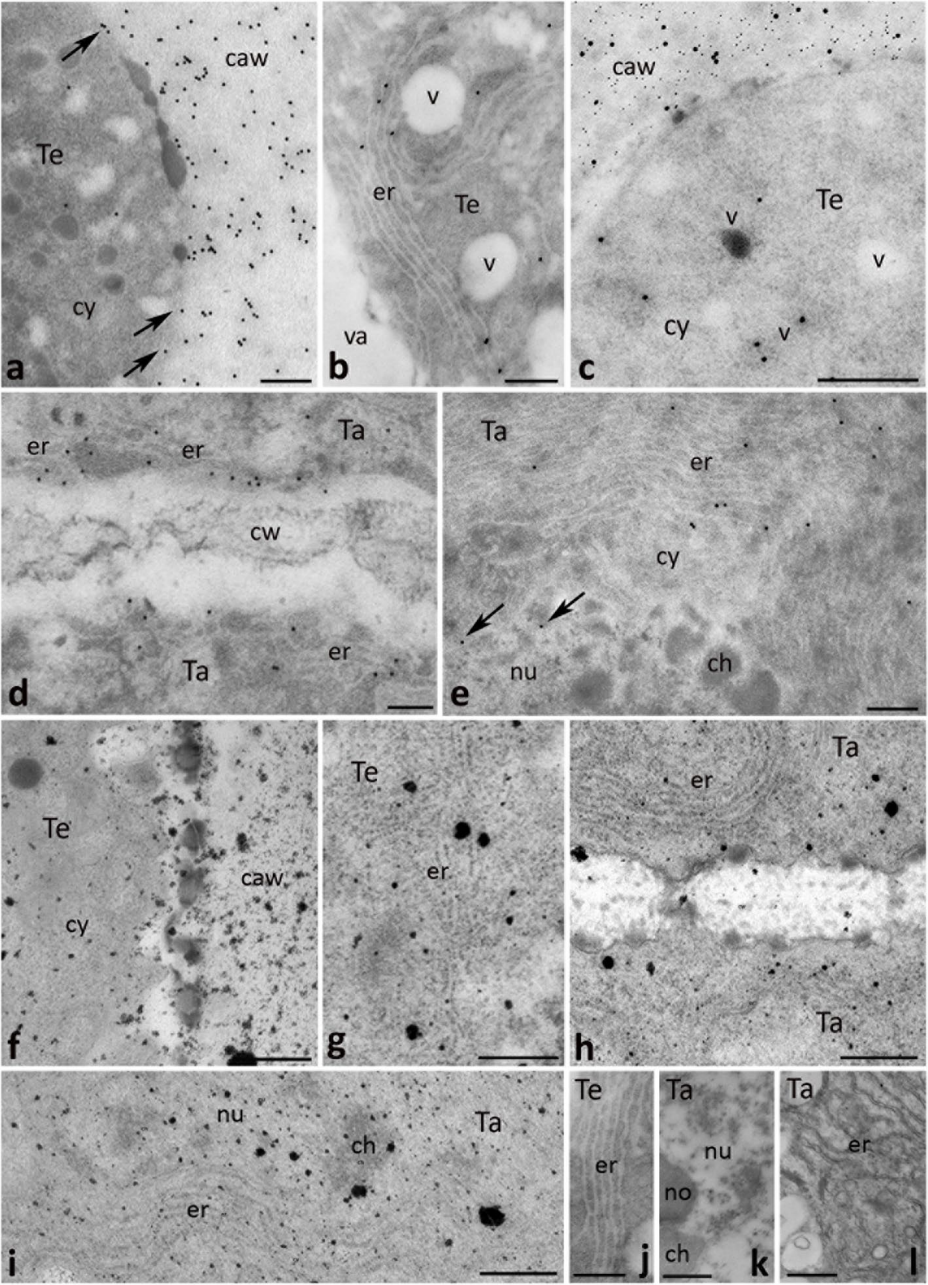
Cross sections via the single locule of *Petunia* anther at the tetrad stage (*Te*) show: immunogold localization of CRT protein (**a**, **b**, **d**, **e**), double labeling (**c**) of CRT protein (20 nm gold particles) and callose (10 nm gold particles), localization of exchangeable Ca^2+^ by potassium antimonite precipitation (**f**-**i**), and the control of immunolabeling (**j**, **k**) and potassium antimonite precipitation (**l**). *Arrows* in **a** show immunolabeling at cell peripheries of meiocyte close to callosic cell wall (*caw*), and *arrows* in **e** show immunolabeling associated with perichromatin areas. *ch* chromatin, *cw* cell wall, *cy* cytoplasm, *er* endoplasmic reticulum, *no* nucleolus, *nu* nucleus, *Ta* tapetum cell, *v* vesicle. *Scale bars* correspond to 500 nm

During meiosis (Fig. 5a), CRT was frequently present in the cytoplasm and nuclei of the dyads and tetrads. Within the meiocyte cytoplasm, CRT labeling was evident in the ER (Figs. 5b, 6b), while in the nuclei CRT PAb were associated mainly with perichromatin areas (Fig. 5c). At this stage of microsporogenesis, some gold traces were found in the cortical cytoplasm of the dyads (Fig. 5d, arrows) and tetrads (Fig. 6a, arrows), and several epitopes binding CRT PAb were also identified in the thick cell wall that surrounded dyads (Fig. 5d). By contrast, numerous gold traces were observed in the cell wall that surrounded tetrads (Fig. 6a). Double labeling demonstrated that extracellular CRT localization in tetrads was tightly correlated with callose deposition (Fig. 6c). It should be noted that CRT staining in the callosic cell wall was predominantly associated with several electron-dense patches (Fig. 6a); they were present in both the meiocyte cytoplasm (Fig. 6c) and the callose deposition (Fig. 6a, c). During meiosis, we found a very specific CRT localization in the tapetal cells, where we observed numerous gold traces in the ER (Fig. 6d, e), including the ER adjacent to the cell peripheries (Fig. 6d). Similar to the previous stage, Ca^2+^ ppts were observed in the same localizations where CRT was found. There was the ER in meiocytes (Figs. 5e, 6g) and the tapetal cells, including the ER adjacent to the peripheral cytoplasm (Fig. 6h), and to the nuclear envelope (Fig. 6i). Ca^2+^ ppts were also detected in the nuclei of meiocytes (Fig. 5f) and the tapetal cells (Fig. 6i) as well as at the cell peripheries and callosic wall surrounding meiocytes (Figs. 5g, 6f). The control sections in which no CRT PAb/potassium antimonate was used were devoid of labeling (Fig. 6j, k and l, respectively).

**Fig. 7:**
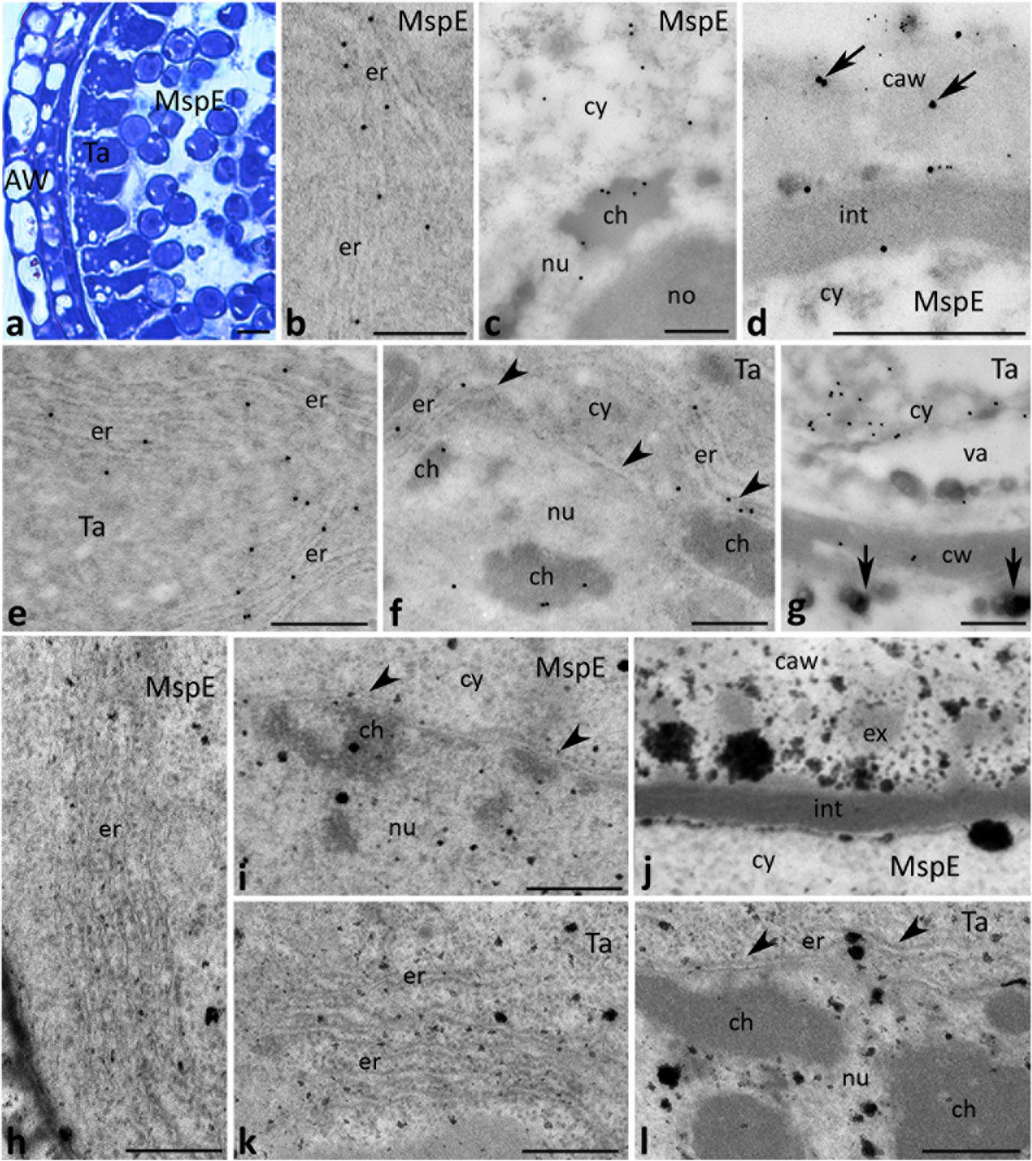
Cross sections via the single locule of *Petunia* anther at the early microspore stage (*MspE*) show: methylene blue staining (**a**), immunogold localization of CRT protein (**b**, **c**, **e**-**g**), double labeling (**d**) of CRT protein (20 nm gold particles) and callose (10 nm gold particles), and localization of exchangeable Ca^2+^ by potassium antimonite precipitation (**h**-**l**). *Arrows* in **d** show CRT labeling in the callosic cell wall (*caw*), *arrows* in **g** show orbicules, and *arrowheads* in **f**, **i** and **l** show nuclear envelope. *Aw* anther wall, *ch* chromatin, *cw* cell wall, *cy* cytoplasm, *er* endoplasmic reticulum, *ex* exine, *int* intine, *no* nucleolus, *nu* nucleus, *Ta* tapetum cell, *va* vacuole. *Scale bars* correspond to 20 μm (**a**) and 500 nm (**b**-**l**)

**Fig. 8:**
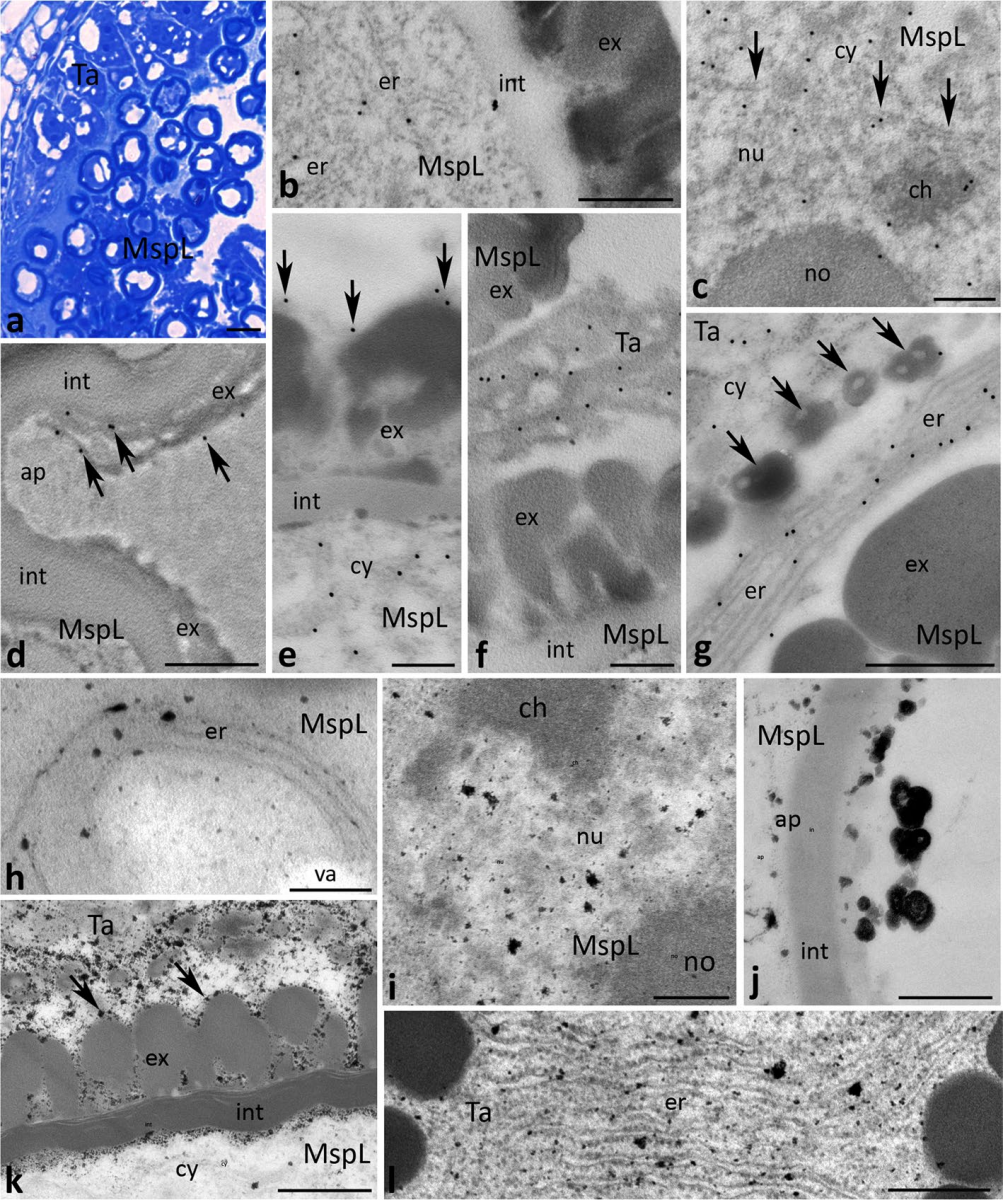
Cross sections via the single locule of *Petunia* anther at the late microspore stage (*MspL*) show: methylene blue staining (**a**), immunogold localization of CRT protein (**b**-**g**) and localization of exchangeable Ca^2+^ by potassium antimonite precipitation (**h**-**l**). *Arrows* in **c** show nuclear envelope, *arrows* in **d** and **e** show CRT labeling associated with the residual tapetum/pollen coat, *arrows* in **g** show orbicules, and *arrows* in **k** show Ca^2+^ ppts associated with the residual tapetum/pollen coat. *ap* aperture, *ch* chromatin, *cy* cytoplasm, *er* endoplasmic reticulum, *ex* exine, *int* intine, *no* nucleolus, *nu* nucleus, *Ta* tapetum cell, *va* vacuole. *Scale bars* correspond to 20 μm (**a**) and 500 nm (**b**-**l**)

After meiosis, the localization patterns of CRT differed in the early and late microspore stages due to the lysis of the callose cell wall at the early microspore stage (Fig. 7a), and the onset of tapetum degeneration at the late microspore stage (Fig. 8a). Intracellularly, the protein was localized mainly in the ER (Figs. 7b and 8b) and nuclei (Figs. 7c and 8c) of microspores. Some gold traces were also found on the border between the cytoplasm and nucleoplasm, adjacent to the nuclear envelope (Fig. 8c, arrows). By contrast, we found clear differences in the localization of CRT in both the tapetum and the extracellular spaces. At the early microspore stage, just few gold traces were detected in the disintegrating cell wall around the microspores (Fig. 7d, arrows). Double-labeling experiments using both CRT PAb and a monoclonal antibody against callose (Cal MAb) clearly showed that CRT co-localized tightly with callose deposition. In tapetal cells, CRT labeling often corresponded with the position of the ER within the cytoplasm (Fig. 7e-g), including the ER adjacent to the nuclear envelope (Fig. 7f, arrowheads). Epitopes binding CRT PAb were also identified in the tapetal cell nuclei (Fig. 7f). In the tapetum showing early signs of disintegration at the late microspore stage, CRT was predominantly observed in the ER enriched the tapetal cytoplasm adjacent to maturing microspores (Fig. 8f, g). Some gold traces were also detected in the extracellular space, including the residual tapetum deposited on the exine layer of the pollen sporoderm (Fig. 8d, e, arrows). It should be noted that no CRT labeling was detected in orbicules, the unique sporopollenin particles secreted by tapetum at the early and late microspore stages (Figs. 7g and 8g, respectively, arrows). As we expected, at this phase of pollen development numerous Ca^2+^ ppts were found in the ER present in the early/late microspores (Figs. 7h and 8h) and the tapetal cells (Figs. 7k and 8l), including the ER adjacent to the nuclear envelope (Fig. 7l, arrowheads). They were also observed in the nuclei (Figs. 7i, l and 8i) and extracellularly, in the disintegrating callosic cell wall (Fig. 7j) or degenerating tapetum (Fig. 8k), including the residual tapetum (Fig. 8k, arrows). At the late microspore stage, numerous Ca^2+^ ppts were also associated with the aperture region of the microspore (Fig. 8j).

**Fig. 9:**
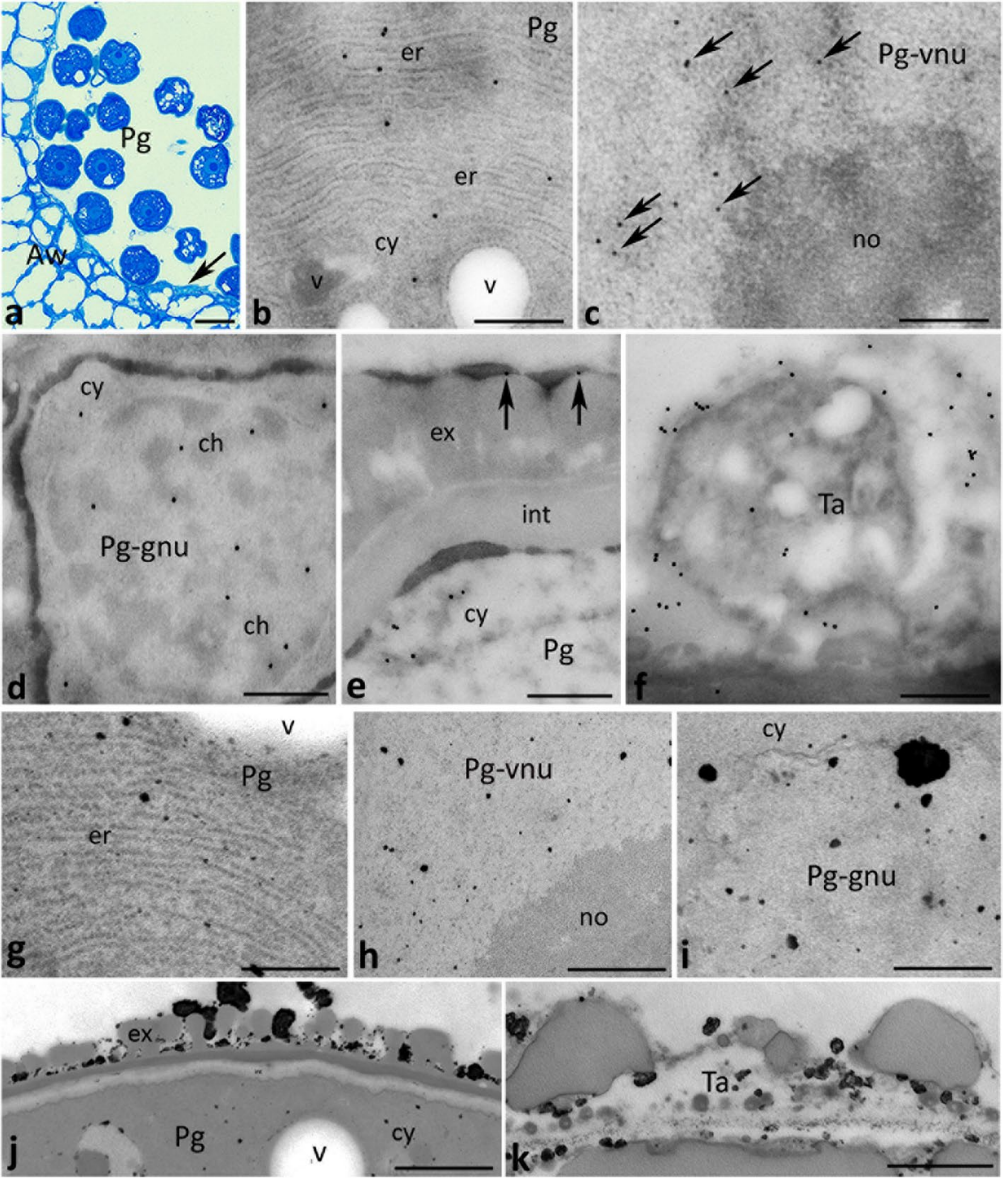
Cross sections via the single locule of *Petunia* anther at the pollen grain stage (*Pg*) show: methylene blue staining (**a**), immunogold localization of CRT protein (**b**-**f**) and localization of exchangeable Ca^2+^ by potassium antimonite precipitation (**g**-**k**). *Arrow* in **a** shows degenerating tapetum, *arrows* in **c** show CRT labeling in the chromatin associated with the nucleolus, and *arrows* in **e** show CRT labeling associated with the residual tapetum/pollen coat. *ch* chromatin, *cy* cytoplasm, *er* endoplasmic reticulum, *ex* exine, *int* intine, *no* nucleolus, *Pg-gnu* generative nucleus of the pollen grain, *Pg-vnu* vegetative nucleus of the pollen grain, *Ta* degenerating tapetum, *v* vesicle. *Scale bars* correspond to 25 μm (**a**) and 500 nm (**b**-**k**)

Finally, we investigated subcellular localizations of CRT and exchangeable Ca^2+^ at the pollen grain stage (Fig. 9a). Intracellularly, both CRT and Ca^2+^ ppts were found in the ER-rich cytoplasm of maturing pollen (Fig. 9b and g, respectively). In addition, the presence of CRT we confirmed in perichromatin areas of the vegetative nucleus of the pollen grain (Fig. 9c, arrows), while the nucleolus was devoid of the labeling (Fig. 9c). Some gold traces were also found in the generative nucleus of the pollen grain (Fig. 9d). Both nuclei were positive for Ca^2+^ labeling (Fig. 9h, i), but no Ca^2+^ ppts were detected in the nucleolus of the vegetative nucleus (Fig. 9h). Extracellularly, both CRT and Ca^2+^ ppts were present at the residual tapetum (Fig. 9e, arrows, and j, respectively). In addition, numerous gold traces and Ca^2+^ ppts were localized to the degenerated tapetum (Fig. 9f, k, respectively) that was visible occasionally at the last stage of pollen development (Fig. 9a, arrow).

Taken together, we conclude that Ca^2+^ ppts corresponding to exchangeable Ca^2+^ were observed in the same localizations where CRT was found during subsequent stages of microsporo/gametogenesis in developing *Petunia* anther.

## Discussion

Previous work from our laboratory provided evidence that CRT may be involved in Ca^2+^ homeostasis and molecular chaperoning during the key reproductive events in *Petunia* pistil, such as pistil transmitting tract maturation, pollen-pistil interactions, double fertilization, and early embryogenesis [17, 18, 22]. We also demonstrated that CRT has a critical role in pollen tube growth in vitro [20]. The importance of CRT in these processes suggests that this protein play some important roles during complicated communications of developing male gametophyte with highly specialized sporophytic cells in the anther, such as the tapetal cells. Here we report that CRT is highly expressed in *Petunia* anther and its localization pattern correlates with loosely bound Ca^2+^ during microsporo/gametogenesis. Our present results provide support for both of the main functions of CRT – regulation of Ca^2+^ homeostasis and molecular chaperoning – during the multi-step process of pollen development in angiosperms.

The angiosperm anther is a male reproductive organ whose development is strictly regulated. To date, our understanding of the biological role of Ca^2+^ in sexual reproduction of higher plants is mainly focused on its essential role in pollen germination and pollen tube elongation. Although characteristics of Ca^2+^ distribution patterns during the anther development have been studied in several plant species, the role of this factor and molecular mechanism/s of regulation of Ca^2+^ homeostasis during pollen development is still poorly understood. Ca^2+^ exists in cells in three main forms, each required for specific activities in cellular metabolism and physiological functions [23]: (1) insoluble covalently bound Ca^2+^ plays mainly a structural role; (2) cytosolic free Ca^2+^ acts as a second messenger in multiple signal transduction pathways; and (3) loosely bound (exchangeable) Ca^2+^ is associated with fixed and mobile anions and is in dynamic equilibrium with the free Ca^2+^. Exchangeable Ca^2+^ is sequestered in different organelles or cell compartments, such as ER and the cell wall, where it can be associated with specific proteins that buffer and release Ca^2+^ to control its local concentration. This mechanism suggests an important role of Ca^2+^-binding/buffering proteins in stabilization of Ca^2+^ homeostasis in living cells. A good candidate for providing this function is CRT.

Here we have demonstrated that both CRT and exchangeable Ca^2+^ have similar distribution patterns during pollen development in *Petunia* anther. This is the first study to fully characterize the sites of localization of these two factors during the complicated intercellular communication that occurs between the developing male gametophyte and the sporophytic cells in the anther. First, we confirmed a permanent presence of CRT and Ca^2+^ ppts in the cytoplasm of the germ line and tapetal cells during the successive stages of microsporo/gametogenesis. The importance of exchangeable Ca^2+^ in the regulation of anther development has often been suggested for years. For example, Tian et al. [24] found that numerous Ca^2+^ ppts accumulate within tapetum and locules of rice anthers, but only few in developing microspores. Dynamic localization patterns of exchangeable Ca^2+^ were also observed in anthers of tabacco [25], lettuce [26] and oil tea [27]. In wheat, this pool of Ca^2+^ has been suggested to control the formation of vacuoles from mitochondria to regulate microspore development [28]. In addition, sterile anthers display abnormal distribution of Ca^2+^ ppts compared to fertile anthers in male sterile plants [24, 29]. These findings indicate that developing anthers require certain amounts of exchangeable Ca^2+^ in specific times and positions during pollen development, and that its abnormal distribution is related to pollen abortion. Second, our immunogold and cytochemical experiments revealed that both CRT and Ca^2+^ ppts preferentially localizes to the ER of these cells. The ER is known to be the most effective intracellular Ca^2+^ store in eukaryotic cells [30], whereas CRT is a Ca^2+^-binding/buffering protein typically residing in the ER lumen [7]. Because CRT is able to bind and sequester Ca^2+^ with high affinity and capacity, it can serve as a mobile intracellular store of easily releasable Ca^2+^ and control its local concentration within the cytoplasm. Third, we detected the epitopes binding CRT PAb and Ca^2+^ ppts at plasmodesmata connecting adjacent microsporocytes or adjacent tapetal cells as well as connecting microsporocytes with the tapetal cells. It has long been recognized that plasmodesmata of higher plants contain a central strand of tightly compressed ER that creates the cytosolic sleeve providing continuity of cytoplasm between adjacent cells [31]. Thus, the specific linear localization patterns of CRT and exchangeable Ca^2+^ at plasmodesmata connecting adjacent cells in *Petunia* pollen chambers most likely corresponds with the ER position.

A key piece of data in support of our idea is our finding that the *PhCRT1* gene, highly expressed in *Petunia* anther, encodes a CRT1 isoform belonging to the CRT1/2 subgroup. These isoforms work as primary proteins within a general ER chaperone framework related to regulation of Ca^2+^ homeostasis [8–10]. Instead, CRT3 isoform does not have high-capacity Ca^2+^-binding ability [32] suggesting functional specialization of plant CRTs. Our current results are consistent with the previous studies by Christensen et al. [15] who reported that *CRT1/2* genes were highly expressed in *Arabidopsis* developing/mature anther, whereas the expression of *CRT3* gene was limited to mature anther/pollen. We previously revealed that the *PhCRT* gene expressed in *Petunia* pistil also belongs to the *CRT1/2* subclass [17]. We showed an elevated expression of *PhCRT1/2* during pistil transmitting tract maturation, pollen-pistil interactions, double fertilization, and early embryogenesis [17] when the level of exchangeable Ca^2+^ changed dynamically [18]. We also demonstrated that *Petunia* pollen tubes growing in vitro accumulated of both *PhCRT1/2* mRNA and CRT protein in the ER-rich regions [19], where CRT activity is required for stabilization of the tip-focused Ca^2+^ gradient in elongated pollen tubes [20] Finally, we found that siRNA-mediated silencing of *PhCRT1/2* expression strongly impairs pollen tube growth [20]. Therefore, based on our previous and current studies we propose a critical role for CRT1 isoform in stabilization of Ca^2+^ homeostasis during pollen development in *Petunia* anther. This idea seems to be supported by results obtained by Yu et al. [33]. These authors characterized a Ca^2+^-binding protein, OsDEX1, which is expressed in tapetal cells and microspores during early anther development in rice. In *osdex1* mutant anther, tapetal cell degeneration is delayed and degradation of the callosic cell wall surrounding the microspores is compromised, leading to aborted pollen formation and complete male sterility. Recombinant OsDEX1 is able to bind Ca^2+^ and regulate Ca^2+^ homeostasis in vitro, and *osdex1* exhibited disturbed Ca^2+^ homeostasis in tapetal cells. These findings confirm a fundamental role of Ca^2+^-binding proteins in the development of tapetal cells and pollen formation, possibly via modulating the Ca^2+^ homeostasis during pollen formation.

One of our most interesting observations was that CRT was concentrated in the callosic cell wall surrounding dyads and tetrads. Accumulation of the CRT PAb in the callose deposition begins with the first meiotic division, peaking in the tetrad stage, and then disappears as the callosic cell wall is lysed after meiosis. This localization pattern of CRT corresponds well with the high level of loosely bound Ca^2+^ in the same compartment, strongly suggesting that this pool of Ca^2+^ is bound by CRT. Since CRT has typical ER targeting and retention signals, it resides primarily in the ER lumen [7]. However, localization of this protein outside the ER has also been observed in eukaryotic cells. Plant CRT was detected in many different compartments and structures, including dictyosomes, vesicles, protein bodies, nucleus, plasma membrane, and the cell wall [22]. Although, some previous work from our lab and others indicated location of CRT in several extracellular regions were somewhat controversial, an elegant work of Luczak et al. [34] provided clear evidence that CRT, similar to several other proteins, is present in the cell walls of few plant species including maize, *Lupinus* and *Arabidopsis*. We have previously demonstrated that both CRT and exchangeable Ca^2+^ were localized to the extracellular peripheries of highly specialized plant cells, such as pollen tubes and synergids [14, 16, 18, 22]. In pollen tubes, CRT labeling was detected in the peripheral ER-rich cytoplasm of the tube adjacent to the cell membrane and in the callosic cell wall. In this extracellular compartment, CRT was usually associated with electron-dense patches and numerous Ca^2+^ ppts were found in extracellular peripheries of elongated pollen tubes. Our present results have revealed the same localization patterns for both CRT and exchangeable Ca^2+^ in the callosic cell wall surrounding dyads and tetrads during microsporogenesis in *Petunia* anther. Moreover, our double labeling experiments using the CRT PAb and monoclonal antibody against (1 → 3)-β-glucan (Cal MAb) have confirmed that CRT co-localized with callose in this specific cell wall. Based on our previous and present data, we cannot exclude the possibility that this external Ca^2+^ store is equally important during the male gametophyte development as the main internal store, which is the ER. It should be noted that the neutral polymer of (1 → 3)-β-glucan does not bind Ca^2+^. Thus, we postulate that excess of exchangeable Ca^2+^ is translocated from the cell cytosol not only to the ER but also to the callosic cell wall, and bound by CRT present there in order to maintaining Ca^2+^ homeostasis in meiocytes during microsporogenesis. We proposed this idea previously when we observed co-localization of CRT, callose and Ca^2+^ ppts in the internal cell wall of growing pollen tube [14, 16, 22]. More precise research is required, however, to verify this hypothesis.

It is commonly accepted that CRT acts as a Ca^2+^-binding/buffering lectin-like molecular chaperone involved in proper folding, quality control, and maturation of newly synthesized glycoproteins within the secretory pathway [7]. The *Petunia* anther has the most common type of tapetum in angiosperms – the secretory (glandular) type. During microsporogenesis, the specialized tapetal cells remains around the anther locule. They exhibit highest secretory activity from the tetrade stage until the late microspore stage, and then they start breaking down just before the anther dehiscence. Tapetal cells play a vital role by secreting proteins, signals, and pollen wall material to ensure microspore/pollen development. In general, secretory cells are characterized by very high rate of protein synthesis and numerous ER cisternae and dictyosomes in close association with the plasma membrane. We have observed similar ultrastructure in the *Petunia* tapetal cells, in which CRT was highly abundant. Our immunogold experiments further revealed that CRT preferentially localizes to the ER, which is prominent in the cytoplasm of the tapetal cells during microsporogenesis. Thus, we suggest that in addition to regulating Ca^2+^ homeostasis, the high level of CRT expression in tapetum reflects a role for CRT as a chaperone to achieve high secretory activity of this highly specialized tissue to ensure pollen development. In fact, our immunoblots revealed a gradual increase in CRT level from the microsporocyte stage through the meiosis; the highest CRT level was at the microspore stage, when both microspores and tapetal cells show extremely high secretory activity correlated with the biogenesis of the sporoderm. By contrast, the highest level of *PhCRT1* mRNA was observed at the microsporocyte stage; expression then decreased gradually through the subsequent stages of pollen development and dropped significantly in dry pollen. Together, these results indicate that *PhCRT1* transcription in *Petunia* anther starts before meiosis, whereas CRT translation is postponed and reaches maximum level at the microspore stage, when the highest rates of protein synthesis and protein folding in the ER may be strictly required. These results are partially consistent with the previous studies by Nardi et al. [13] in *Nicotiana* anther who reported the expression of *CRT* mRNA throughout microsporogenesis until the mature pollen stage, when the level of *CRT* mRNA significantly decreased. However, these authors detected the strongest immunohistochemical signal using the anti-spinach CRT antiserum in both the germ line and tapetal cells at the tetrad stage, when the sporoderm biogenesis starts. Regardless of these slight differences, the obtained results support the hypothesis that CRT’s chaperone activity may facilitate the high rate of protein synthesis required for pollen development.

Interestingly, CRT was also detected in the lipid-rich tapetosomes that are abundant organelles in secretory tapetum of *Brassica* anther [21]. At an early stage of the anther development, the tapetal cells possess abundant rough ER and secretory vesicles, apparently for active exocytosis of molecules into the locule for pollen development and maturation. Next, the tapetal cells become packed with two abundant and predominant storage organelles, the elaioplasts and the tapetosomes. At a late stage of the anther development, the tapetal cells lyze, and their content is discharged to the locule and deposited onto maturing pollen, forming the pollen coat. These authors revealed that CRT existed in tapetal cells during early anther development [21]. Subsequently, CRT appeared together with oleosins in the ER network. Finally, the ER network largely disappeared, and solitary tapetosomes containing CRT and oleosins became abundant. After tapetum cells lyzed, however, oleosins but not CRT of tapetosomes were transferred to the pollen surface. By contrast, our results clearly showed that CRT was predominantly observed in the ER enriched the tapetal cytoplasm adjacent to maturing microspores, but some gold traces were also detected in the anther locule, including the residual tapetum deposited on the pollen sporoderm. As we expected, at the late phase of pollen development numerous Ca^2+^ ppts were found in the same localizations, including the ER of degenerating tapetum and the residual tapetum. Both CRT and Ca^2+^ ppts were also found in the apertures of maturing pollen grains. Therefore, further investigations are needed to explain CRT’s role at the late stage of pollen development within the anther.

Finally, we report the presence of CRT in the nuclei of several gametophytic and sporophytic cells in *Petunia* developing anther, such as microsporocytes, dyads/tetrads, microspores, pollen grains, and the tapetal cells. The immunogold research have shown highly selective CRT distribution in specific nuclear sub-domains, especially in the nuclei of the tapetal cells with dispersed chromatin and large nucleoli. In these cells, we have detected CRT within chromatin, including the perichromatin areas and the nucleolus-associated chromatin. Several theories could explain the functional importance of CRT within the nucleus. Perichromatin fibrils are the sites of transcription and co-transcriptional processing of pre-mRNA and maturation of pre-RNA/mRNA [35]. Therefore, CRT associated with the nuclear sub-domain may be involved in regulating of these important nuclear processes. We found that CRT’s localization in the nuclei and nuclear envelope of different cell types within *Petunia* anther corresponded with sites of exchangeable Ca^2+^. This could indicate that CRT modulates nuclear Ca^2+^ homeostasis by regulating the local Ca^2+^ concentration. The lumen of the nuclear envelope is contiguous with the lumen of the rough ER and can form a branching intra-nuclear network which could serve as a mobile store of Ca^2+^ [36]. Recently, Rivas-Sendra et al. [6] revealed unique Ca^2+^ dynamics (free Ca^2+^ ions) in in vivo rapeseed microspores as well as in those reprogrammed to in vitro embryogenesis, establishing a link between changes in the cytosolic Ca^2+^ level and its nuclear distribution in the male gametophyte. Such dynamic changes in the Ca^2+^ levels between the nucleus and the cytosol require a very efficient and mobile Ca^2+^ store. CRT seems to be an excellent candidate to fulfill this role.

## Conclusions

Our data for the first time clearly show an interplay between CRT1 and exchangeable Ca^2+^ during pollen development in the anther. Based on the obtained results, we strongly believe that CRT1 plays crucial role in regulation of Ca^2+^ homeostasis during formation of the male gametophyte in angiosperms, and its further functioning as a pollen tube. Currently, a role of diverse ER chaperones, including CRT isoforms, in pollen development and the tube formation is under heated debate and opposing arguments are put forward by different research groups. We have recently proved that post-transcriptional silencing of the *PhCRT1/2* expression strongly impairs pollen tube growth [20]. In contrast, Vu et al. [37] indicated that *Arabidopsis* triple mutant of *CRT1/2/3* grows and develops almost normally, while calnexin (*CNX1*) expression is crucial for normal pollen development, pollen germination and the tube elongation. Nevertheless, these authors suggest that both CNX and CRT proteins play essential and overlapping roles during vegetative growth and the male gametophyte development, and their complex relationship is strictly required for normal pollen formation, pollen germination and the tube elongation. It has to be noted, however, that the recent work published by Wakasa et al. [38] has shown that silencing of the endogenous *CNX* genes does not have any impact for obtaining viable progeny in rice. Therefore, future functional studies are needed to help us sort out these possibilities.

## Methods

### Plant material

Commercial cultivars of *Petunia hybrida* were grown at room temperature in the Department of Cellular and Molecular Biology, Faculty of Biological and Veterinary Sciences, Nicolaus Copernicus University in Toruń, Poland. Fresh anthers of *Petunia* were dissected from flowers at different developmental stages: microsporocytes, dyads/tetrads, microspores, and pollen grains. To study callose metabolism during microsporogenesis, germ line cells were isolated mechanically from one of five anthers of the same flower, stained with 0.1% aniline blue according to a standard protocol, and observed under a fluorescence microscope. This staining was helpful in determining particular developmental stages of microsporogenesis. For northern hybridization and western blot analysis, dissected anthers and dry pollen collected from flowers at the anther dehiscence were frozen in a liquid nitrogen and stored at − 80 °C until they were used. For light and electron microscopy, dissected anthers were fixed, dehydrated in ethanol, and embedded according to the protocols described below. To analyze the histological features of the anther at each of the developmental stages, semi-thin sections of the embedded anthers were stained with 0.1% methylene blue and observed by light microscopy. All experiments described below were repeated at least three times during several growing seasons with similar results.

### Synthesis of the molecular probes and northern hybridization

Two digoxigenin (DIG)-labelled molecular probes, the full-length *PhCRT1* antisense probe and the *Ph18S* probe, were prepared as described previously [17]. In brief, purified plasmid DNA containing the *PhCRT1* insert was cut with Xba I and purified on CHROMA SPIN™ + TE − 100 columns. A 1-μg aliquot served as template to generate the DIG-labeled molecular probe using the DIG RNA Labeling Kit (SP6/T7) according to the manufacturer’s instructions (Roche). To obtain DIG-labeled *18S* probe, plasmid containing the internal part of the *Ph18S* rRNA sequence was used as template. The probe was generated by PCR according to the manufacturer’s instructions (PCR DIG Synthesis Kit, Roche).

Northern hybridization was performed according to the protocol described previously [17]. In brief, plant material (samples of 100 mg) was ground in liquid nitrogen. Total RNA was extracted in RNA Extracol (EURx) according to the manufacturer’s protocol, RNA concentrations were measured, and RNA quality was assessed by visualization on a 1.2% agarose gel. A 15-μg aliquot of total RNAs were dried under a vacuum, resuspended in 5 μl of RNase-free H_2_O, supplemented with two volumes of denaturing buffer containing 50% formamide, 6.1% formaldehyde, 1 × MOPS, 1 × loading dye buffer, and 40 mg/dm^− 3^ ethidium bromide, and heat denatured. RNA samples were resolved on a 1.2% formaldehyde-agarose gel, capillary transferred onto Immobilon-NY^+^ membrane (Millipore), rinsed with H_2_O mQ, and UV crosslinked. The membrane was prehybridized for 40 min in DIG Easy Hyb buffer (Roche) at 65 °C, and then hybridized overnight to the DIG-labeled *PhCRT11* probe at a final concentration 150 ng/ml. Chemiluminescence detection was performed according to the manufacturer’s instruction (DIG Luminescent Detection Kit, Roche). To reprobe the membrane with *Ph18S* DIG-labeled probe, the blot was stripped twice at 80 °C in 50% formamide, 5% SDS, 50 mM Tris–HCl, pH 7.5, for 60 min, and then rinsed with 2 × SSC. Rehybridization was performed in the same conditions as hybridization described above. Northern hybridization was performed a minimum of three times, and representative blots were shown. Quantification of signals was done with Image Gauge 3.4 software (Science Lab99). All data obtained from the northern blot experiments were subjected to one-way ANOVA test.

### Western blot analysis

Immunoblotting was performed according to the protocol described previously [18]. In brief, plant material (100 mg of samples) *was* homogenized in liquid nitrogen, and soluble proteins were extracted in 50 mM Tris–HCl (pH 7.5), 1 mM EGTA, 2 mM DTT plus 1 mM PMSF and cOmplete Protease Inhibitor Cocktail (Roche) according to the manufacturer’s recommendation. The homogenates were centrifuged at 16,000 *g* for 30 min at 4 °C. Protein concentrations of the supernatants were measured, equal amounts of proteins were separated by electrophoresis on a 12.0% SDS–PAGE gel, and then the proteins were semi-dry transferred to Amersham PVDF Hybond-P membrane (GE Healthcare). Blocked blot was probed with a rabbit polyclonal antibody CRT PAb produced by Napier et al. [39], washed, and probed with the antibody against rabbit IgG conjugated with horseradish peroxidase (HRP, Merck). Signal was detected with the Amersham ECL Advance Western Blotting Detection Kit according to the manufacturer’s guidelines (GE Healthcare). Next, the membrane was stripped and re-probed with a primary antibody against glyceraldehyde-3-phosphate dehydrogenase (mouse anti-GAPDH IgG, Abcam). Detection was performed as described above using anti-mouse HRP-conjugated secondary antibody. Immunoblotting was performed a minimum of three times, and representative blots were shown. Quantification of signals was done with Image Gauge 3.4 software (Science Lab99). Statistical significance of data was determined by a one-way ANOVA test.

### Fluorescent in situ hybridization

FISH was performed according to the protocol described previously [17] with slight modifications. Selected anthers were fixed with freshly prepared 4% (*v/v*) formaldehyde and 0.25% (*v/v*) glutaraldehyde in phosphate-buffered saline (PBS, pH 7.2) for 1 h at room temperature (slight vacuum infiltration) followed by overnight fixation at 4 °C. The fixed samples were washed in PBS, dehydrated through a graded series of ethanol, embedded in LR Gold resin (Fluka), sectioned into semi-thin sections (cross sections through the anther), and transferred onto microscope slides covered with Biobond (BBInternational). The DIG-labeled antisense *PhCRT1* molecular probe (generated above) was used at a final concentration of 0.5 μg/μl. Prehybridization and hybridization were carried out in 50% formamide, 4 × SSC, 5 × Denhardt’s, and 50 mM sodium phosphate buffer for 1 h at 42 °C and overnight at 37 °C, respectively. Signals were detected using primary mouse anti-DIG (Roche) and secondary goat-anti-mouse IgG-Alexa Fluor 488 (Life Technologies) antibodies in PBS. For negative control, the molecular probe was omitted. In the final step, DNA was stained with 1 mg/ml Hoechst 33342 (Molecular Probes) and the stained sections were mounted in ProLong Gold antifade reagent (Life Technologies). The sections were analyzed using a Nicon Eclipse 80i microscope. FISH was performed a minimum of three times, and representative data were shown.

### Immunocytochemistry

Immunocytochemical localization of CRT was performed according to the protocols described previously [14, 18, 22] with slight modifications. Selected anthers were fixed and embedded as described above. Semi-thin and ultra-thin sections (cross sections through the anther) were transferred onto microscope slides covered with Biobond (BBInternatioanal) or collected on Formvar-coated nickel grids, respectively. After blocking with 3% (*w/v*) bovine serum albumin (BSA) in PBS buffer, pH 7.2, the sections were incubated in 1:20 dilution of a primary CRT PAb in PBS supplemented with 0.3% (*w/v*) BSA and then incubated with: (i) a goat anti-rabbit IgG Cy3® secondary antibody (Sigma-Aldrich) for immunofluorescence labeling; (ii) a goat anti-rabbit IgG 1 nm gold-conjugated (BBInternational) secondary antibody for immunogold-silver staining technique; (iii) a goat anti-rabbit IgG 20 nm gold-conjugated (BBInternational) secondary antibody for immunogold labeling. For CRT and callose immunolocalizations, a double labeling technique was performed as previously described [14, 22]. In brief, after blocking with 3% BSA, ultra-thin sections were treated with two kinds of primary antibodies: CRT PAb and monoclonal anti-(1 → 3)-β-glucan antibody (Cal MAb, Biosupplies). Signals were detected with the following secondary antibodies: 20 nm gold-conjugated goat anti-rabbit IgG (for CRT) and 10 nm gold-conjugated goat anti-mouse IgG (for callose), both from BBInternational. Finally, semi-thin sections were stained with 1 mg/ml Hoechst 33342 (Molecular Probes) or treated with Silver Enhancing Kit for Light and Electron Microscopy (BBInternational) according to the manufacturer protocol. Stained sections were mounted in ProLong Gold antifade reagent (Life Technologies) and analyzed using a Nicon Eclipse 80i microscope. The ultra-thin sections were stained with 2.5% uranyl acetate and examined on a Jeol EM 1010 transmission electron microscope. For negative control, the primary CRT PAb was omitted. Each of the immunocytochemical experiment was performed a minimum of three times, and representative data were shown.

### Potassium antimonate precipitation

Localization of exchangeable Ca^2+^ was performed according to the protocol described previously [18, 22]. In brief, selected anthers were fixed with freshly prepared 2% (*w/v*) potassium antimonate, 2% (*v/v*) glutaraldehyde, and 2% (*v/v*) formaldehyde in 0.1 M phosphate buffer (KH_2_PO_4_, pH 7.8) for 4 h at room temperature, and then subsequently postfixed with 1% (*v/v*) osmium tetroxide (OsO_4_) in the same buffer-antimonate solution for 12 h at 4 °C. The fixed samples were dehydrated in graduated ethanol concentrations and embedded in Spurr resin (Merck) according to the standard protocol. Ultra-thin sections (cross sections through the anther) were collected on copper grids, stained with 2.5% (*w/v*) uranyl acetate and 0.4% (*w/v*) lead citrate solutions, and examined by transmission electron microscopy (Jeol EM 1010) at 80 kV. The presence of Ca^2+^ in the Ca^2+^-antimonate precipitates (Ca^2+^ ppts) was confirmed previously using energy-dispersive X-ray microanalysis [40]. For negative control, potassium antimonate was omitted during fixation. Potassium antimonate precipitation was performed a minimum of three times, and representative data were shown.

## Supplementary Information

**Additional file 1: Figure S1.** Northern blot analysis (**a** and **b**) and western blot analysis (**c** and **d**) in whole *Petunia* anthers during subsequent stages of pollen development (*Msc* microsporocyte, *Dy/Te* dyad/tetrad, *Msp* microspore, *Pg* pollen grain stages) and in dry pollen (*Pd*). **a** *PhCRT1* mRNA, **b** *Ph18S rRNA*, **c** *Ph*CRT, **d** *Ph*GAPDH (*boxed*). Numbers above each blot point out separate lines; **M** protein marker (Protein Marker VI, Applichem). The top and bottom blot edges are marked with *solid lines*.

## Data Availability

The datasets used and/or analyzed during the current study available from the corresponding author on reasonable request.

## Abbreviations

Ca^2+^: Calcium ions
Ca^2+^ ppts: Ca^2+^-antimonate precipitates
Cal Mab: Monoclonal anti-(1 → 3)-β-glucan antibody
*CRT*/CRT: Calreticulin gene/protein
CRT PAb: Polyclonal antibody against CRT
ER: Endoplasmic reticulum
FISH: Fluorescent in situ hybridization
