# Supplementary figures and images for: Calreticulin expression and localization in relation to exchangeable Ca2+ during pollen development in Petunia
Source: BMC Plant Biol. 2022 Jan 8;22:24. doi: 10.1186/s12870-021-03409-4 (PMC8742381; doi:10.1186/s12870-021-03409-4)

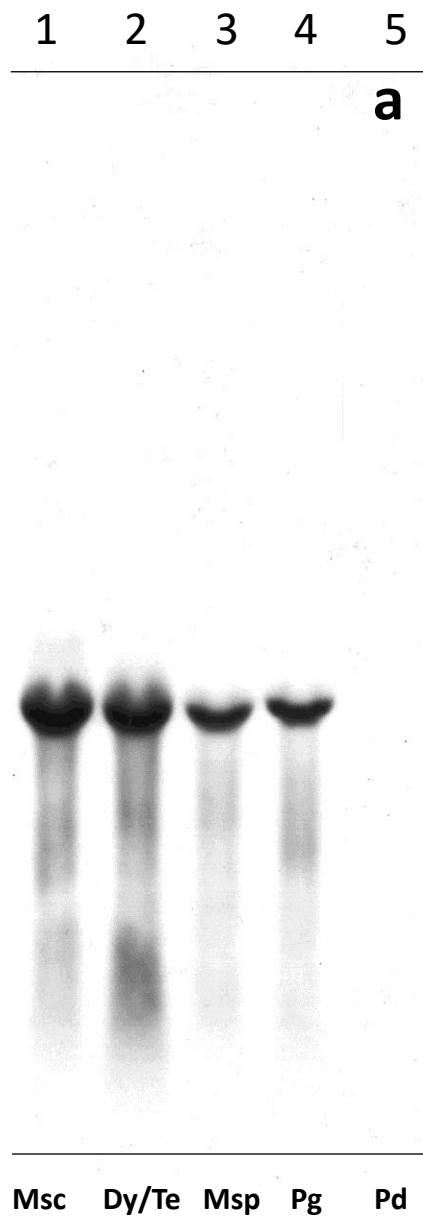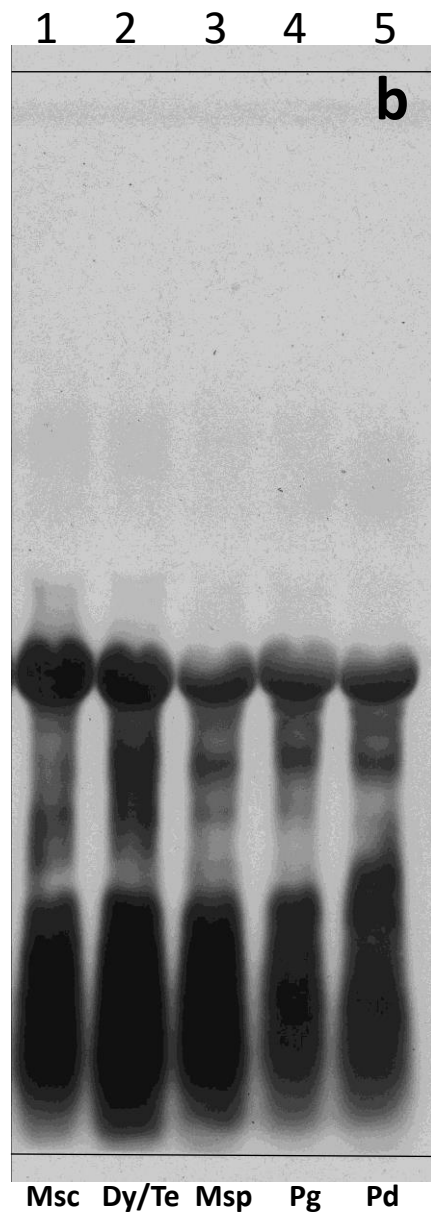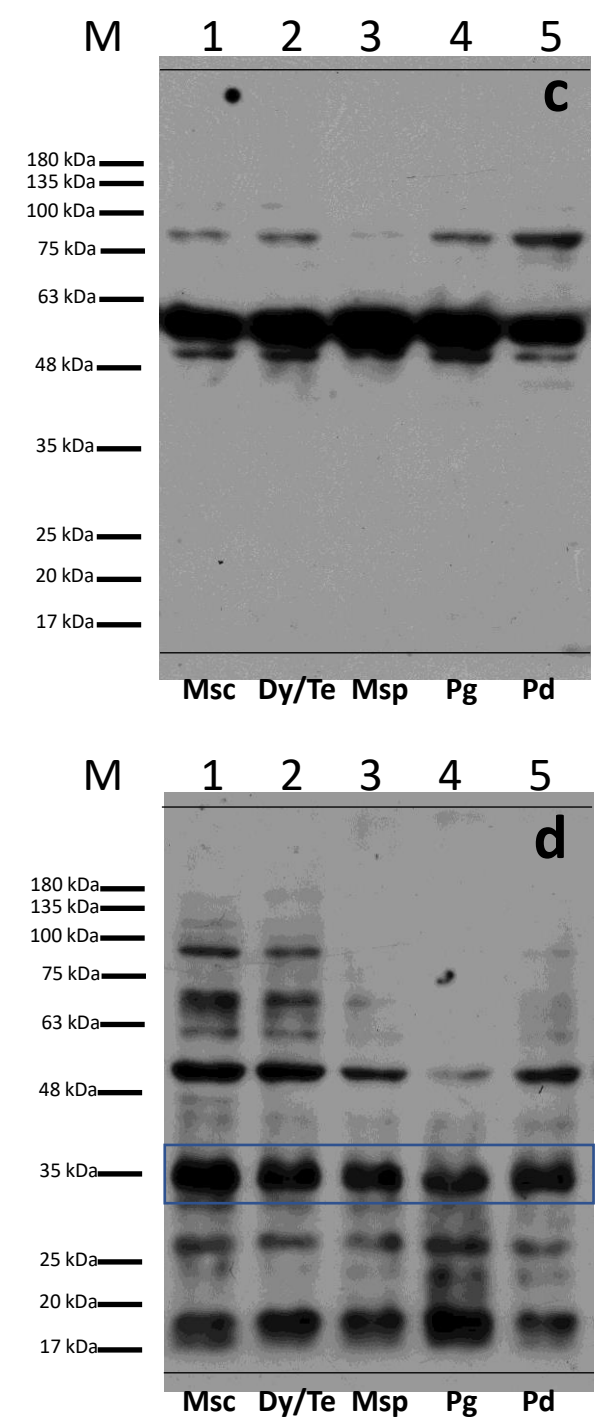

Supplement: Supplementary file 1 — Additional file 1: Figure S1. Northern blot analysis (a and b) and western blot analysis (c and d) in whole Petunia anthers during subsequent stages of pollen development (Msc microsporocyte, Dy/Te dyad/tetrad, Msp microspore, Pg pollen grain stages) and in dry pollen (Pd). a PhCRT1 mRNA, b Ph18S rRNA, c PhCRT, d PhGAPDH (boxed). Numbers above each blot point out separate lines; M protein marker (Protein Marker VI, Applichem). The top and bottom blot edges are marked with solid lines. [file 12870_2021_3409_MOESM1_ESM.pdf]
